# Malaria and malnutrition in tribal areas of India: implications for paediatric health

**DOI:** 10.3389/fgstr.2025.1526806

**Published:** 2025-05-14

**Authors:** Anil Kumar Verma, Suyesh Shrivastava, Nishant Saxena

**Affiliations:** ^1^ Division of Vector Borne Diseases, ICMR-National Institute of Research in Tribal Health, Jabalpur, M.P., India; ^2^ Academy of Scientific and Innovative Research, AcSIR Headquarters, Ghaziabad, India; ^3^ Division of Non-Communicable Diseases, ICMR-National Institute of Research in Tribal Health, Jabalpur, M.P., India; ^4^ Division of Social Sciences, ICMR-National Institute of Research in Tribal Health, Jabalpur, M.P., India

**Keywords:** malaria, malnutrition, tribal, India, water safety and hygiene (WASH), pediatric health

## Abstract

Malaria and malnutrition (undernutrition) are associated with poor health and poverty. Undernutrition affects children’s general development, particularly the formation of a healthy immune system. Malaria parasites infect and propagate within vulnerable populations, causing anemia, fever, and, if ignored, death. Furthermore, frequent exposure to malaria and intestinal infections alters gut flora, exacerbating malnutrition caused by poor digestion and malabsorption of ingested food. In India, Tribal people are trapped in a vicious cycle of hunger and infectious diseases, such as malaria, due to inadequate diet, unsanitary practices, water quality, sanitation, attitude, and lack of access to healthcare facilities. Therefore, an integrated approach that caters to the nutritional and malaria-specific needs of the tribal population is required to eliminate malaria from tribal areas of India. This mini-review discusses the problems and probable solutions for the control and elimination of malaria in the tribal areas in India.

## The nexus of malaria and malnutrition

Millions of individuals in tropical and subtropical nations are affected by malaria, a fatal parasitic disease caused mostly by *Plasmodium falciparum*, *Plasmodium vivax*, *Plasmodium malariae*, and *Plasmodium ovale.* Occasionally, *Plasomdium knowlesi* infects humans, although its primary target is primates. WHO Africa (94%) and WHO Southeast Asia regions contributed more than 95% of malaria cases in 2023. Outside Africa, India is a major contributor of malaria. In recent years, the efforts of the Indian government have come to fruition, and India has been able to exit high-burden high-impact (HBHI) countries in 2024 after successfully reducing the number of malaria cases and deaths (1). Although malaria and malnutrition form a common link between India and WHO Africa, the epidemiology of malaria differs vastly. On the one hand, *P. falciparum* is responsible for most malaria cases in Africa, but in India, four species of malaria parasite have been recorded, with *P. falciparum* and *P. vivax* contributing more than 95% of cases (2). This double health burden of malaria and malnutrition (undernutrition) is a common public health problem plaguing both countries. The geographical, demographic, and socioeconomic distributions of malaria and malnutrition largely overlap across the tropical and subtropical regions of low-income and middle-income countries, including India and Africa (3, 4). In India, malaria and malnutrition are mainly concentrated in the rural and tribal areas of the country.

## The burden of malaria in tribal populations of India

**Figure 1:**
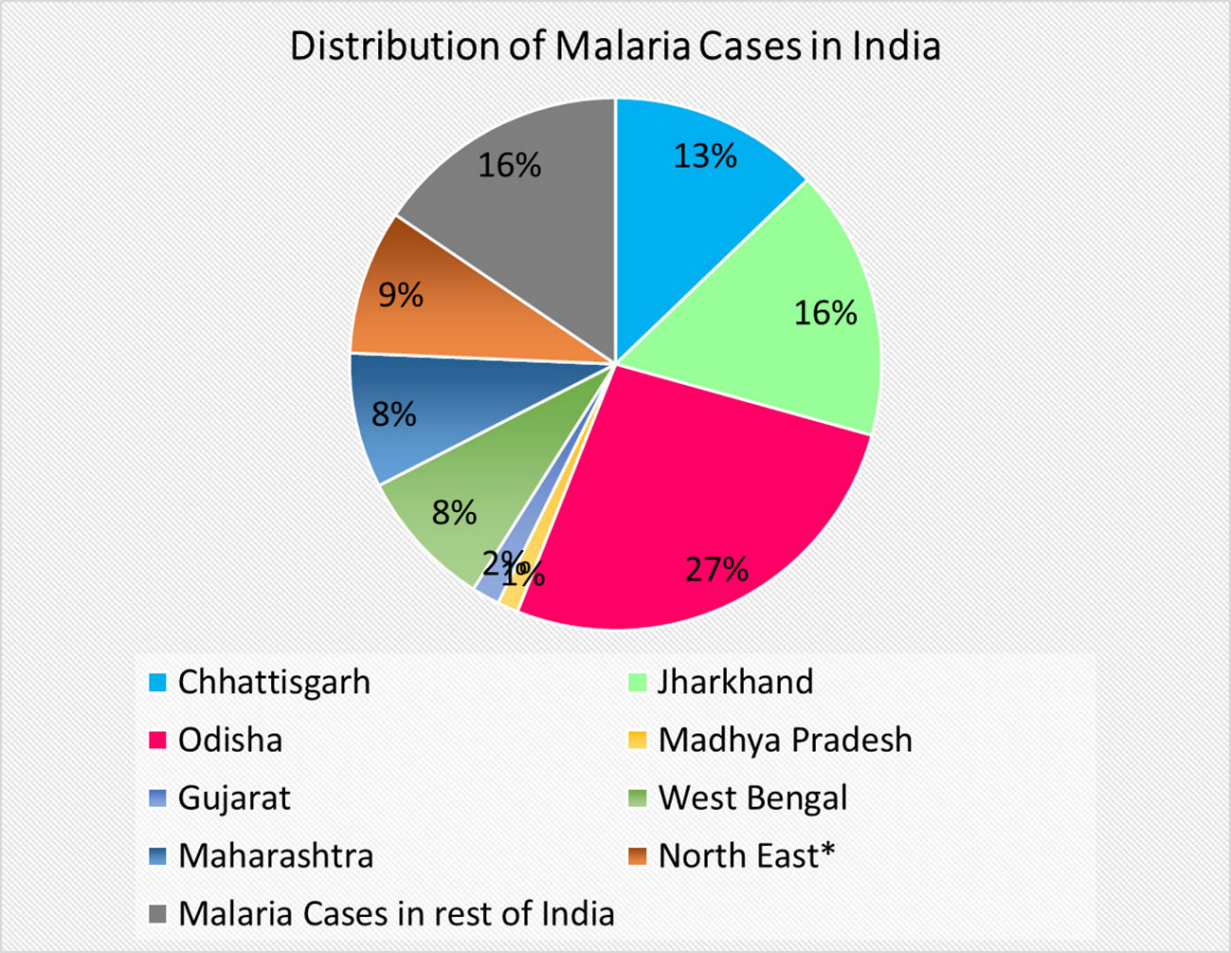
Pie chart showing distribution of malaria in Tribal dominated states of India and rest of India. *North east states include Arunachal Pradesh, Assam, Manipur, Meghalaya, Mizoram, Nagaland, Sikkim.

The tribal population, which accounts for only 8.6% of the total population of India, is mostly distributed in seven states (Odisha, Jharkhand, Madhya Pradesh, Maharashtra, Chhattisgarh, Gujrat, West Bengal) and the northeastern region (NER consists of Arunachal Pradesh, Assam, Manipur, Meghalaya, Mizoram, Nagaland, Tripura, and Sikkim). Most of these states are dominated by indigenous (tribal) people (the highest in Mizoram (94.44%) to the lowest in Uttar Pradesh (0.57%)), who suffer the most from malaria. This tribal population represents 43.32% of the total population of India and contributes to more than 80% of the total malaria cases in the country (5). In 2024, these states contributed 84.48% of the cases to total malaria cases in India (**Figure 1**) (NVBDCP, 2025) (6).

## The victims of malaria and malnutrition

Children below age 5 are the main victims of malaria and malnutrition. In 2023, children less than 5 years of age contributed to 73.7% of deaths due to malaria, whereas nutritional issues account for nearly 50% of mortality in children (under the age of five) worldwide (1, 7–9). The immune system in malnourished children is largely compromised by T cell dysfunction and reductions in neutrophil microbicidal activity, the number of dendritic cells, antigen priming and presentation, and protein levels in the complement cascade (10). Subsequently, children with severe acute malnutrition are at a significant risk of death from infectious illnesses, such as pneumonia, diarrhea, and malaria. Furthermore, malaria and malnutrition (undernutrition) mutually help each other in the malaria-endemic tribal areas. The percentage of anemia in malaria-stricken children is often high, and malnourished children are at high risk of malaria infection. Furthermore, tribal populations are economically disadvantaged, undernourished, and live in rural areas with poor water quality, sanitation, and hygiene (WASH). It has been recently observed that malnutrition and anemia are important confounders that influence malaria epidemiology in the endemic states of central and eastern India (11).

## The “vicious cycle” of malaria, malnutrition, and poor WASH

Malnutrition affects children’s general development (12). As a result of malnutrition, there is impairment of the skin, respiratory, and gut barriers that facilitate infectious diseases. The triad of malaria, malnutrition, and the environment (WASH) creates a vicious cycle of vulnerability in which the tribal population is trapped. Infections due to inadequate WASH conditions lead to morphological changes in the villus (villus blunting and atrophy), malabsorption, mucosal inflammation, and alterations in gut flora (12, 13). Furthermore, multiple studies have shown that Plasmodium infections may alter the gut microbial flora of the host, influencing the clinical outcome of malaria (14, 15). Malnutrition is exacerbated by changes in the gut flora and villus structure, which lead to poor digestion and malabsorption of ingested food.

A recent study reported very high prevalence of stunting (39%), wasting (157%), and anemia (60%) among children in India (16). Moreover, malnutrition in the tribal communities was higher than the national average. Approximately 44% of the tribal children are stunted, 45% are underweight, and 27% are wasted (NFHS-4). One of the major clinical causes of malnutrition in pregnant women is the birth of a low-weight baby. Furthermore, despite having parasitemia, severely malnourished children do not show the typical symptoms (fever) of malaria early in comparison to healthy children (17). These severely malnourished cases of asymptomatic malaria may facilitate transmission in the community as a reservoir. Moreover, malnourished children with uncomplicated malaria have a higher risk of delayed parasite clearance, ACT treatment failure, and reinfection (4). The manifestation of clinical symptoms of malaria is the result of intricate interactions between hosts, parasites, and the environment. Apart from partial acquired immunity, the coexistence of malnutrition and poor WASH conditions in malaria-endemic areas is probably the root cause of variable clinical presentation of malaria from asymptomatic to severe malaria, leading to death.

## Poor health infrastructure and health seeking behavior

Furthermore, in tribal areas, healthcare and transportation infrastructure are poor. It has been observed that the average distance of any government health care facility (primary health center or community health center) varies by 13 km and 28 km or more, respectively, from tribal villages (18). In a recent study conducted in the Chhindwara District of Madhya Pradesh, it was observed that during any illness, the majority of tribal communities (~64%) approached Unlicensed Medical Practitioners (49%) and faith/herbal healers (15%) for their health care needs. Only a small section (30% of households) sought treatment from the public healthcare system (18). Additionally, the availability of private hospital care is limited and expensive in the tribal areas. These healers are unaware of the national malaria treatment guidelines and administer medications without a proper diagnosis. Integrating them into the public health system is difficult because of their substandard education. However, they may be incentivized to channel tribal communities to public healthcare systems to meet their health needs. This will help in the prompt diagnosis and correct treatment of malaria and other diseases.

## Learnings from tribal areas

The UN Sustainable Development Goals (SDGs-2) are to end hunger and malnutrition by 2030, and the Government of India targets the elimination of malaria by 2030 (19). However, nutrition-sensitive schemes and malaria elimination programs work in Indian silos. Recently, two important malaria control studies were conducted, namely Durgama Anchalare Malaria Nirakarana (DAMaN) in Odisha (20) and Mandla-Malaria Elimination Demonstration project (M-MEDP) in Madhya Pradesh (21). The main aim of these studies was to demonstrate the feasibility of malaria elimination in malaria-endemic areas using the currently available tools and resources. MEDP focused on 1. Micro-level Implementation: Targeted interventions at the village level led to a drastic reduction in malaria cases. 2. Early Diagnosis & Treatment (EDT): Strengthened surveillance systems ensure timely diagnosis and treatment, and reduce transmission. 3. Vector Control Measures: Indoor residual spraying (IRS), use of insecticide-treated nets (ITNs), and environmental management play crucial roles in reducing mosquito populations. 4. Community Engagement: Active involvement of local communities and ASHA workers ensured better awareness and adherence to preventive measures. 5. Technology Integration: Use of digital tools for real-time tracking and monitoring of malaria cases improved the response efficiency. 6. Collaboration with Private Sector: Partnerships with private entities such as the Foundation for Disease Elimination and Control of India (FDEC-India) brought additional expertise and funding. (21, 22).

Durgama Anchalare Malaria Nirakaran (DAMaN)—Odisha focused on 1. Reaching Remote Areas: Focused on malaria-prone tribal and inaccessible regions, ensuring healthcare access where previously limited. 2. Mass screening and treatment: Conducted mass screening of communities provided treatment to all confirmed cases, significantly reducing parasite reservoirs. 3. Long-Lasting Insecticidal Nets (LLINs): LLINs are distributed on a large scale, leading to a decline in mosquito bites and malaria transmission. 4. Community Mobilization: Local participation and awareness programs helped ensure the acceptance and sustainability of interventions 5. Sustained Government Commitment: Strong political will and state-level prioritization led to sustained funding and policy support. 6. Nutritional status assessment and nutritional supplementation (20).

Both programs focused on robust surveillance (for early case detection and prompt treatment to break the transmission cycle using intensive track, test, treat, and track strategy), vector control measures (such as the distribution of long-lasting insecticidal net and indoor residual spraying), and community mobilization and health awareness using information, education and communication/behavior change communication (IEC/BCC) as community participation enhances the success and sustainability of malaria control efforts. Targeted interventions in high-risk areas (tribal, remote, and forested regions) are essential for malaria elimination. The use of technology improves data accuracy, surveillance, and response time. Moreover, government and private sector collaboration accelerates the impact and scalability of elimination. However, the DAMaN also assessed the nutritional status of the target group (pregnant/lactating women and children aged <5 years). Anemic mothers were provided with iron and folic acid supplementation whereas children were provided with nutritional support thorough Integrated Child Development Services (ICDS).

## Multisectorial coordination and community engagement

Several nutrition-sensitive schemes of the Government of India under the multi-ministerial convergence of POSHAN Abhiyaan (Anganwadi Services, Public Distribution System (PDS), Pradhan Mantri Matru Vandana Yojna, Swachh Bharat Mission, Mid-day meal scheme) are committed to addressing the challenge of persistent malnutrition in India. However, the implementation and penetration of these schemes in tribal areas are challenging. Despite several schemes targeting maternal and child healthcare, the uptake by tribal communities is very low. Tribal areas that are particularly vulnerable to malaria require intricate multi-sectoral coordination and interventions. Programs to eradicate malaria and malnutrition must be integrated because of their co-occurrence and complementary relationships with these diseases in the tribal communities. In India, health is a state subject; therefore, Indian states need to step up to counter the challenge of malaria and malnutrition through a diversified and locally suited strategy for vulnerable tribal populations. Community awareness and engagement are of the utmost importance. There is a need for a well-designed socially sensitive ICC/BCC strategy for awareness generation and promotion of healthcare-seeking behavior among tribal communities, coupled with the implementation and promotion of nutrition-sensitive schemes in tribal areas. Every affected district can develop an integrated digital dashboard for monitoring malaria disease, nutritional vulnerability, and supply, stock, and distribution of resources through a simplified mobile app. The development of transportation and telecommunication facilities in tribal areas will improve accessibility and communication (23). Further, tribal communities need to engage in work opportunities created by the Mahatma Gandhi National Rural Employment Guarantee Act (MNREGA). Since several factors such as biological, environmental, social, structural, and economic increase vulnerability to malaria, the involvement and contribution of all stakeholders are crucial for the elimination of malaria in tribal areas.

## The need of the hour

**Figure 2:**
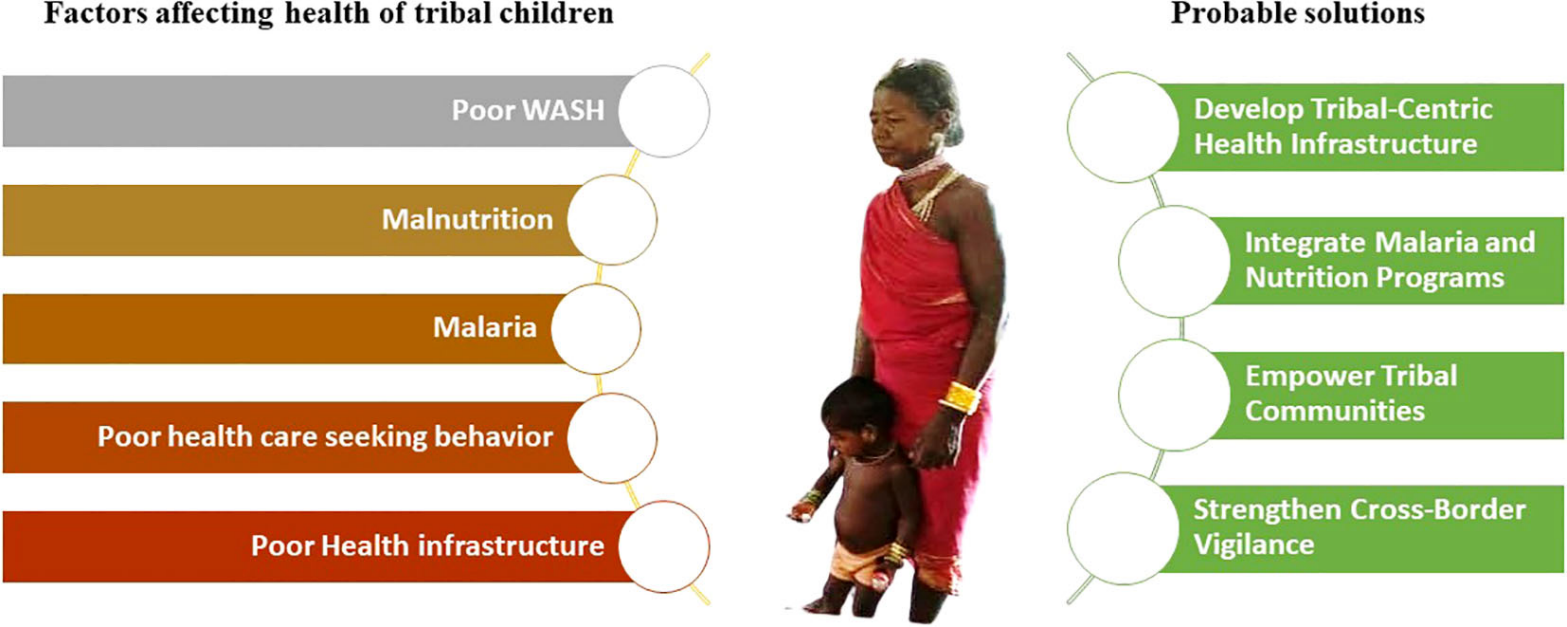
Schematic representation of various factors affecting the health of tribal children and probable solutions to uplift the tribal health.

Tribal communities in India are at a critical juncture. Malaria and malnutrition are not mere health challenges; they are intertwined crises that perpetuate poverty, hinder development, and threaten lives in the most vulnerable populations. The time for incremental steps is over; bold, integrated action is nonnegotiable. Malaria cases have dropped by 80% since 2015, yet hotspots such as Mizoram, Tripura, Chhattisgarh, Odisha, Jharkhand, and Maharashtra persist, fuelled by cross-border transmission, drug resistance, and inadequate surveillance in remote tribal areas. Simultaneously, malnutrition ravages these communities, with 46.5% stunting in Meghalaya, rising wasting in Nagaland, and anemia crippling generations. These are not isolated issues: malaria thrives where malnutrition weakens immunity, and malnutrition deepens where malaria drains vitality. We urge to 1. Prioritize Tribal-Centric Health Infrastructure: Deploy mobile health units, trained ASHA workers, and drone-delivered supplies to reach forested, hilly terrains where roads end, but needs do not. 2. Integrate Malaria and Nutrition Programs: Combine vector control (LLINs, IRS) with nutrition drives—fortified foods, micronutrient supplements, and clean water—to break the vicious cycle 3. Strengthen Cross-Border Vigilance: Collaborate with Bangladesh and Myanmar to curb malaria importation, a silent saboteur in the northeast.4. Empower Communities: Invest in tribal education and local health champions to tackle cultural barriers, teenage pregnancies, and sanitation gaps that fuel malnutrition. 5. Accelerate Funding: Redirect resources to high-burden districts—do not let bureaucratic delay cost lives (**Figure 2**). India’s 2030 goals—malaria elimination and zero hunger—hinge on the tribal heartlands. Act decisively, act now, or risk leaving millions behind. The data are clear and the solutions are known. What is needed is an unwavering resolution. Tribal people deserve no less. We emphasize urgency, integration, and accountability tailored to the unique challenges of tribal regions, such as those in the northeast. Until an integrated and sustained effort to address the challenges of malaria and malnutrition, the elimination of malaria from tribal areas will remain an illusion in India.
